# Evaluation of the effects of occlusal splint and masseter muscle injection in patients with myofascial pain: a randomised controlled trial

**DOI:** 10.22514/jofph.2024.028

**Published:** 2024-09-12

**Authors:** Reyhan Saglam, Cagri Delilbasi, Gulsum Sayin Ozel, Irmak Durur Subasi

**Affiliations:** ^1^Department of Oral and Maxillofacial Surgery, School of Dentistry, Istanbul Medipol University, 34083 Istanbul, Turkey; ^2^Department of Prosthodontics, School of Dentistry, Istanbul Medipol University, 34200 Istanbul, Turkey; ^3^Department of Radiology, International School of Medicine, Istanbul Medipol University, 34200 Istanbul, Turkey

**Keywords:** Masseter muscle, Masticatory muscle disorders, Occlusal splint, Shear wave elastography, Temporomandibular disorders, Trigger point injection

## Abstract

Myofascial pain is one of the common symptoms in patients with temporomandibular joint disorders (TMD). Occlusal splint (OS) and masticatory muscle trigger point (TP) local injections are primary treatment options. We aimed to investigate the effects of these treatments using clinical and elastography measures. Patients who were diagnosed with myofascial pain according to Diagnostic Criteria for Temporomandibular Disorders (DC/TMD) were included. There were 16 patients in each group. Group 1 was treated with occlusal splint, Group 2 was treated with occlusal splint and masseter muscle lidocaine injection, Group 3 was treated with masseter muscle lidocaine injection and Group 4 consisted of healthy volunteers. Degree of pain and maximum mouth opening (MMO) were recorded. Masseter muscle stiffness was evaluated by Shear wave elastography. Measurements were repeated at 1st and 3rd months of post-treatment. Pain decreased at all times in all the patients (*p* = 0.001). Pain in Group 2 and Group 3 approached 0 level at 3rd month. MMO increased from baseline to 1st month and from 1st month to 3rd month and masseter stiffness decreased from baseline to 1st month and to 3rd month (*p* = 0.001) in all groups. Occlusal splint and masseter muscle lidocaine injection were effective in reducing pain and increasing MMO in patients with myofascial pain. All treatments reduced masseter muscle stiffness. All the treatment modalities had clinically similar and successful outcomes.

## 1. Introduction

Myofascial pain is one of the most important symptoms in patients with temporomandibular joint disorders (TMDs). Although the etiological factors causing TMD cannot be defined precisely, conditions such as bruxism, parafunctional habits, stress and anxiety may cause inflammation in the joint capsule and pain in the masticatory muscles [1, 2]. Myofascial pain, temporomandibular joint clicking, limitation in mouth opening, headache and even psycho-social disorders are among the symptoms of TMDs [1]. TMDs can be caused by irregularities and disorders in the joint area (intra-articular), and in the surrounding muscular structures (extra-articular).

Trigger points (TPs) are one of the important clinical signs of myofascial pain. It contains a sensory component of sensitized nociceptors and leads to pain perception. Patient’s self-complaints and muscle tenderness on palpation are important clinical symptoms in the diagnosis of masticator motor system dysfunction which is seen in approximately 90% of patients [3]. Patients diagnosed with myofascial pain commonly refer to clinics sustaining stiffness of the masticatory muscles, pain and limitation in mouth opening [4, 5, 6, 7].

Treatment options of myofascial pain are quite complex and often vary depending on the patient symptoms. These include, occlusal splints (OS), supportive patient exercises, interventions to reduce stress and anxiety, muscle exercises, postural modifications, drug treatments (such as nonsteroidal anti-inflammatory drugs, myorelaxants, benzodiazepines, selective serotonin reuptake inhibitors), acupuncture and dry needling [1]. Local anesthesia without vasoconstrictor and botulinum toxin injections are also some of the primary treatment options. Among these options, OS therapy and TP injections are effective in reducing pain. They also increase mouth opening and maximum bite force [1, 8].

Ultrasonography can detect changes in the viscoelastic properties of soft tissue, and correlation between muscle structure and function can be better interpreted by ultrasonography [9, 10]. Various elastography techniques have been used to assess muscle stiffness. Shear wave elastography (SWE) produces low-frequency shear waves inside tissues and generates sources of mechanical vibration [1]. These waves propagate through the soft tissues and promote their degradation in accordance with the degree of tissue stiffness. These waves are recorded by a scanner. The actual elastic modulus of the region of interest (ROI) can be determined and its stiffness (in kilopascal, kPa) can be recorded. Measurement of muscle stiffness by sonoelastography is a valid and reliable method [11, 12]. SWE has been recently introduced to clinical practice. It is appropriate and reliable technique for evaluation of masseter muscle stiffness in healthy individuals and patients with masseter muscle disorders.

Masticatory muscle stiffness, pain and limitation in mouth opening are primary symptoms in myofascial pain. The most common treatment modalities for these cases include intramuscular local anesthetic injections and oral appliances [1, 4, 5, 6, 7, 8].

Muscle stiffness increases in patients with TMD [4, 5, 6, 7]. The relevant muscles may be affected in patients with myofascial pain and muscle elasticity values may increase. In this study, OS and lidocaine injection to masseter muscle TPs and combination of these treatments were performed in patients with myofascial pain. Patients were followed-up for 3 months. Here we aimed to examine the effects of different treatments on masseter muscle stiffness measured by SWE, as well as clinical parameters such as pain and mouth opening.

## 2. Materials and methods

The investigators implemented a prospective randomised controlled clinical study. A total of 198 patients were examined. Patients’ self-report and palpation of the masticatory muscle together with Diagnostic Criteria for Temporomandibular Disorders (DC/TMD): Diagnostic Decision Tree were used in diagnosis [13].

The inclusion criteria were as follows:

• Presence of myofascial pain according to DC/TMD diagnostic decision tree;

• Patients with mild depression and anxiety levels (score below 5 for each questionnaire) as a result of the Patient Health Questionnaire-9 (PHQ-9) and General Anxiety Disorder-7 (GAD-7) questionnaires on the DC/TMD Axis 2;

• Myofascial pain persisting for more than 3 months;

• Having minimum 3 TPs in the masseter muscles on palpation (active or latent);

• No medical or surgical treatment for TMD;

• No history of OS treatment;

• No history of masticatory muscle injection or dry needling;

• Absence of active caries and pulpal lesions;

• No missing teeth other than the third molar.

Exclusion criteria were as follows:

• Intra-articular disorders or degenerative joint disease according to DC/TMD diagnostic decision tree;

• Patients with moderate, moderately severe and severe depression and anxiety levels as a result of the Patient Health Questionnaire-9 (PHQ-9) and General Anxiety Disorder-7 (GAD-7) questionnaires on the DC/TMD Axis 2;

• Presence of active infection in the masseter TP area;

• Being in the mixed dentition period or using of complete dentures;

• Presence of congenital head and neck deformity;

• Allergy to local anesthetics;

• Acute trauma or infections that affected temporomandibular joint;

• Systemic disorders that may affect temporomandibular joint (*e.g.*, rheumatoid arthritis);

• Presence of bleeding disorder, cardiovascular disease, thyroid disease, diabetes, hypertension, renal failure, isolated muscle disease and systemic disorders that may affect the absorption mechanism of local anesthetics (*e.g.*, hepatic, renal failure);

• Being treated for rheumatological and neurological disorders and/or neuropathic pain and/or headache;

• Odontogenic or nonodontogenic pathologies that may cause pain near the region of the temporomandibular joint;

• Neuropathic pain (*e.g.*, trigeminal neuralgia);

• Having a history of trauma in the head and neck region in the last 2 years;

• Presence of malignancy or having undergone head and neck radiotherapy/chemotherapy in the last 2 years;

• Having drug and/or alcohol addiction;

• Having a known psychiatric disorder and using antidepressants in the last 6 months;

• Being under active orthodontic treatment;

• Pregnancy or lactation;

• Having atypical fascial pain and presence of fibromyalgia.

### 2.1 Study design

Clinical examination (CD) and TP injections to masseter muscle (RS) were performed every-time by the same oral and maxillofacial surgeon. A specialist in Department of Prosthodontics (GSO) who was not involved in the examination at baseline and at follow-up delivered and adjusted the OSs. SWE recordings were performed by a specialist in the Department of Radiology (IDS). The examiners who participated in the study did not know which group the patients belonged to.

In the first examination of the patients;

• 10-cm visual analogue scale (VAS-10 cm) was used for pain evaluation. Pain levels were evaluated between 0–10.

• Pain free maximum mouth opening (MMO), unassisted MMO, assisted MMO, maximum right and left lateral movements (RLM, LLM) and maximum protrusion movements (PM) of all groups were measured with the reference of #11 and #41 teeth.

• Masseter muscle elasticity was evaluated by shear-wave elastography before the masseter muscle lidocaine injection and OS treatment (Baseline values).

All patients were given information about the mechanism of TMD and possible risk factors. Clinical measurements and masseter muscle stiffness were recorded on the day 0, before the patients started to use their OSs and before masseter muscle TP injections. After the masseter muscle stiffness had been recorded, the injections to masseter muscle TPs were performed and the patients started to use their splints. Painkillers and muscle relaxants were not prescribed.

Groups were accordingly;

Group 1: These patients were treated with OS.

Group 2: These patients were treated with OS and lidocaine injection to masseter muscle TPs.

Group 3: These patients were treated with lidocaine injection to masseter muscle TPs.

Group 4: These patients were healthy volunteers (control group).

In Group 2 and Group 3, injections to masseter muscle were repeated two more times, on 7th and 14th days. All clinical parameters and masseter muscle stiffness were measured at 1st and 3rd months of post-treatment.

In the control group, SWE scores were recorded on the day they were examined. Clinical data and SWE scores were recorded only once. These healthy individuals were not recalled for control.

### 2.2 Occlusal splint technique

A 3 mm hard plaque resin material (Sx 3 mm plaque, Bioart, Brazil) was prepared on the plaster model with a vacuum plate pressing machine (Plastvac P7, Bioart, São Carlos, SP, Brazil). The OS with canine ramps was prepared after determining the centric relationship with the anterior stopper. Temporary acrylic resin (Protemp 4, 3M ESPE, Germany) was used to close the 3–4 mm gap between the posterior teeth. The OS was adjusted for simultaneous and equal contact of all anterior and posterior teeth. The acrylic canine prominence was made up for guidance during the protrusive and laterotrusive movements of the mandible. The OSs were delivered to the patients after the adjustments and polishing at their second visit. Patients were instructed to use the splint at night during sleep for three months. Patients were recalled for splint control one week after delivery.

### 2.3 Injection technique

After cleansing the skin, the site of maximum tenderness was identified within the masseter muscle. Injection to TPs was made after the patient agreed the localization of the most painful 3 TPs on right and left sides. The TP was held and stabilised between two fingers (index and middle fingers). 0.3 mL of a plain local anesthetic (H001, Jetokain Simplex, ADEKA, Samsun, Turkey) was injected to each TP. A 30 Gauge (1/2′′ 0.3 × 13 mm) 13 mm MicrolanceTM 3 needle (Becton Dickinson, AG, Fraga, Spain) was used with a 1 mL syringe. After the injection, pressure was applied to the injected area for local hemostasis. As the complaints related to TPs usually continued [14] the injections were repeated 3 times with a 1-week interval, on 7th and 14th days [15].

### 2.4 Shear wave elastography technique

SWE evaluation was performed by using a Logiq E9 Ultrasound Machine (LOGIQ E9 with XDclear, GE Healthcare, Chicago, IL, USA) with a high-frequency linear probe. The patients were in the supine position and were asked not to turn their heads in any direction (neutral position). They were instructed not to grind their upper and lower teeth while lips closed. The stiffness of right and left masseter muscles were investigated. While obtaining the images, approximately 5 mm thick water-based gel (Aquasonic®, Parker Laboratories, USA) was used in order to prevent air retention between the probe and the skin surface and to prevent tissue compression. The probe was contacted with the skin without external pressure, and care was taken that the operator’s hand was not moving. SWE parameters were measured in kPa for elasticity. A 9-MHz transducer of the device was used and the sonoelastography scale was set to 150 kPa with a real-time propagation map. The SWE probe was positioned perpendicular to the anterior border of the right and left masseter muscle and to the underlying mandibular ramus surface. The long axis of the probe was placed parallel to the occlusal plane approximately 15–20 mm above the lower border of the mandible. In the measurements, images were obtained where the color map completely filled the elasticity window and was uniformly and homogeneously distributed. Elasticity map was created in the middle part of the masseter body. Elasticity scores (stiffness) were obtained by placing round ROI with a diameter at least 25 mm^2^ on this map. According to the hardness classification of the device, the red color represented the harder area (increase in elasticity) and the blue color represented the softer area (decrease in elasticity) (Fig. 1).

**Fig. 1.** Evaluation of masseter muscle stiffness by shear-wave sonoelastography.

### 2.5 Post-treatment follow-up

All clinical measurements and masseter muscle stiffness were recorded at the 1st and 3rd months after the treatment. Since 3 patients in Group 1 stated that their pain continued at the end of the 3rd month, OS use was continued. Patients were asked to refer to the clinic again if the pain relapsed.

### 2.6 Statistical analysis

The normal distributions of the data were analyzed using the Shapiro-Wilks test. Numerical data were expressed as mean and standard deviation. One-way Repeated measures analysis of variance model was used for the comparison of continuous variables among baseline, 1st month and 3rd month results.

One-way analysis of variance (One-Way ANOVA) was used for comparison of the variables among the groups. Significantly different groups were determined with the *post-hoc* Tukey test.

The VAS scores among the groups were compared with the Friedman and *post-hoc* Dunn tests. One-way ANOVA was used for the comparison of VAS scores within the groups. Two-way ANOVA was used for the comparison of the variation of VAS scores among the groups.

The correlation between baseline clinical parameters and masseter muscle elasticity was evaluated by Pearson correlation analysis.

*p* value ≤ 0.05 was considered statistically significant. Analyses were performed using SPSS Statistics 23 for Windows (IBM SPSS Inc., Chicago, IL, USA) software.

Power analysis was performed to determine the number of patients in each group.

## 3. Results

It was calculated that a total of 64 individuals, with at least 16 subjects from each group when α = 0.05 and 1 − β = 0.80 were considered in the power analysis (Heinrich-Heine-Universität Düsseldorf, Germany) performed. Due to potential drop outs, 20 patients were recruited for each treatment group.

A total of 198 patients were evaluated for eligibility, 138 of them were excluded from the study. Finally, 60 patients with myofascial pain without referral were included in the study. They were randomly allocated to the treatment groups, which consisted of 20 patients each. Follow-up of a total of 12 patients, 4 from each treatment group, was lost. At the end of the study, the data of 48 patients and 16 healthy individuals, in total 64 individuals were evaluated. Flow chart of the treatment protocol was shown in Fig. 2. In total, only 2 of the patients in Group 2 and 3, who were administered lidocaine injection into the masseter muscle, had a temporary state of facial paralysis. No other complications were encountered.

**Fig. 2.** **Flow chart of the treatment protocol.** TMD: temporomandibular joint disorders.

52 females (81.3%) and 12 males (18.8%) were included in the study. There was no statistically significant difference among the groups in terms of mean age and gender distribution (*p *> 0.05). The mean age of the groups and the gender distribution of the patients regarding the groups are shown in Table 1.

**Table 1.** Baseline findings of the groups.

	n	Female	Male	Age ± SD
Group 1	16	11	5	26.56 ± 4.68
Group 2	16	15	1	27.50 ± 3.48
Group 3	16	13	3	30.13 ± 8.37
Group 4	16	13	3	28.88 ± 6.09

*n: number of patients; SD: standard deviation.*

As a result of the evaluation of the normal distributions, it was determined that the VAS parameter did not show a normal distribution in the groups (*p *> 0.05), while the MMO, RLM, LLM, PM and masseter muscle stiffness parameters fit the normal distribution (*p* < 0.05).

### 3.1 Pain

The mean value of VAS decreased from baseline (the beginning of the treatment) to 1st month and from 1st to 3rd month in all the 3 groups (*p* = 0.001) (Table 2, Fig. 3).

**Table 2.** Statistical comparison of VAS change in the groups.

Group	VAS	n	Mean** ± SD	*p*
Group 1				
	Baseline	16	7.78^a^ ± 1.59	0.001*
	1st month	16	2.53^b^ ± 1.58	
	3rd month	16	0.38^c^ ± 0.47	
Group 2				
	Baseline	16	7.97^a^ ± 1.13	0.001*
	1st month	16	2.69^b^ ± 2.32	
	3rd month	16	0.09^c^ ± 0.20	
Group 3				
	Baseline	16	9.03^a^ ± 1.04	0.001*
	1st month	16	1.00^b^ ± 0.66	
	3rd month	16	0.13^c^ ± 0.22	

Friedman and Post-hoc Dunn tests, *p ≤ 0.05, **Means with different letters show significant difference. n: number of patients; SD: standard deviation; VAS: Visual Analog Scale.

**Fig. 3.** **Comparison of VAS scores within and among the groups.** Group 1: Patients treated with OS, shown in blue. Group 2: Patients treated with OS and lidocaine injection to masseter muscle TPs, shown in red. Group 3: Patients treated with lidocaine injection to masseter muscle TPs, shown in green. Group 4: Healthy volunteers (control group), shown in orange. *p  ≤ 0.05. VAS decreased from baseline to 1st month and from 1st to 3rd month in treatment groups. VAS: Visual Analog Scale.

The mean VAS scores in Group 3 was higher in baseline compared to other groups, however, it was lower than the other two treatment groups at the 1st month. At 3rd month (at the end of the treatment), VAS scores of Group 2, Group 3 and the control group was significantly close to the 0 level (Table 3).

**Table 3.** Comparison of VAS scores among groups.

VAS	Group 1	Group 2	Group 3	Group 4	*p*
	Mean ± SD	Mean ± SD	Mean ± SD	Mean ± SD	
Baseline	7.78 ± 1.59	7.97 ± 1.13	9.03 ± 1.04	0.00 ± 0.00	<0.001*
1st month	2.53 ± 1.58	2.69 ± 2.32	1.00 ± 0.66	0.00 ± 0.00	<0.001*
3rd month	0.38 ± 0.47	0.09 ± 0.20	0.13 ± 0.22	0.00 ± 0.00	0.003*

One-Way ANOVA, *p ≤ 0.05. SD: standard deviation; VAS: Visual Analog Scale.

A statistically significant decrease was observed in Group 3 compared to Group 1 and Group 2 at the end of the 1st month (*p* = 0.001, *p* = 0.001). VAS scores decreased statistically significant from 1st to 3rd months in Group 2 compared to Group 3 (*p* = 0.009). VAS scores decreased statistically more in Group 3 compared to Group 1 at the end of the treatment (*p* = 0.010).

Pain relief in Group 3 was higher at the 1st month and at the end of the treatment compared to the other groups (*p* = 0.010). In Group 3 pain relief in 1st and 3rd months was higher than other groups.

### 3.2 Maximum mouth opening

A statistically significant increase was observed in MMO measurements from baseline to 1st month and from 1st to 3rd month in all treatment groups (*p * ≤ 0.05) (Table 4, Fig. 4a,b, respectively).

**Table 4.** Statistical comparison of MMO values within the groups.

MMO	Group 1 (n = 16)		Group 2 (n = 16)		Group 3 (n = 16)	
	Mean** ± SD (mm)	p*	Mean** ± SD (mm)	p*	Mean** ± SD (mm)	p*
Pain free MMO baseline	39.19^a^ ± 8.14	0.001*	35.81^a^ ± 7.98	0.001*	40.50^a^ ± 6.40	0.001*
Pain free MMO 1st month	44.13^b^ ± 5.58		41.19^b^ ± 5.22		43.72^b^ ± 6.24	
Pain free MMO 3rd month	47.06^c^ ± 6.68		45.88^c^ ± 5.55		45.25^c^ ± 6.65	
Unassisted MMO baseline	42.41^a^ ± 8.24	0.001*	40.06^a^ ± 6.61	0.001*	43.84^a^ ± 6.19	0.001*
Unassisted MMO 1st month	46.75^b^ ± 5.85		43.78^b^ ± 5.58		45.13^b^ ± 6.58	
Unassisted MMO 3rd month	48.47^c^ ± 6.35		46.84^c^ ± 5.57		45.97^c^ ± 6.55	
Assisted MMO baseline	44.94^a^ ± 8.40	0.001*	42.31^a^ ± 6.18	0.001*	43.00^a^ ± 12.12	0.050*
Assisted MMO 1st month	48.44^b^ ± 6.45		45.56^b^ ± 5.45		46.52^b^ ± 6.58	
Assisted MMO 3rd month	49.56^c^ ± 6.25		47.91^c^ ± 5.40		47.19^b^ ± 6.43	

One-Way Repeated measures ANOVA and post-hoc Sidak tests, *p ≤ 0.05, **Means with different letters show significant difference. SD: standard deviation; MMO: Maximum mouth opening.

In control group pain free MMO and unassisted MMO were measured as 42.44 ± 3.24 mm and assisted MMO was measured 43.59 ± 3.01 mm.

There was no statistically significant difference among the groups in the increase in pain free MMO and unassisted MMO from baseline to the 1st month. However, the amount of increase in pain free MMO and unassisted MMO from baseline to 3rd month and from 1st month to the 3rd month, was statistically higher in Group 2 and in Group 3 (*p* < 0.05).

There was no statistically significant difference among the groups in the increase of assisted MMO from baseline to the 1st and 3rd months (Fig. 4c). However, in Group 2 the amount of increase in assisted MMO from 1st to 3rd month was statistically higher than the increase in Group 3 (*p* = 0.01).

**Fig. 4.** **Comparison of Mouth Opening values within and between groups.** (a) Comparison of Pain Free Mouth Opening values within and between groups; (b) Comparison of Unassisted Maximum Mouth Opening values within and between groups; (c) Comparison of Assisted Maximum Mouth Opening values within and between groups. Group 1: Patients treated with OS, shown in blue. Group 2: Patients treated with OS and lidocaine injection to masseter muscle TPs, shown in red. Group 3: Patients treated with lidocaine injection to masseter muscle TPs, shown in green. Group 4: Healthy volunteers (control group), shown in orange. *p  ≤ 0.05. A statistically significant increase was observed in MMO measurements from baseline to 1st month and from 1st to 3rd month in all treatment groups. In Group 3, assisted MMO increased from 1st to 3rd month but it was not statistically significant.

### 3.3 Lateral and protrusive movements

A statistically significant increase was observed in lateral and protrusive movements in treatment groups from baseline to 1st month and from 1st to 3rd month (*p* < 0.05) (Table 5) (Fig. 5a–c). In Group 4, RLM and LLM were measured as 10.84 ± 1.42 and PM was measured as 8.25 ± 1.17.

**Table 5.** Statistical comparison of the lateral and protrusive movements within the groups.

Lateral and Protrusive Movements:	Group 1 (n = 16)		Group 2 (n = 16)		Group 3 (n = 16)	
	Mean** ± SD (mm)	p*	Mean** ± SD (mm)	p*	Mean** ± SD (mm)	p*
RLM baseline	7.91^a^ ± 2.61	0.001*	9.13^a^ ± 1.64	0.001*	8.78^a^ ± 0.66	0.001*
RLM 1st month	9.47^b^ ± 1.54		9.94^b^ ± 1.84		9.59^b^ ± 0.90	
RLM 3rd month	10.28^c^ ± 1.48		10.50^c^ ± 1.71		10.00^b^ ± 1.25	
LLM baseline	9.03^a^ ± 1.74	0.001*	8.59^a^ ± 1.47	0.001*	8.75^a^ ± 0.66	0.001*
LLM 1st month	9.97^b^ ± 1.56		9.69^b^ ± 1.40		9.59^b^ ± 0.86	
LLM 3rd month	10.31^c^ ± 1.41		10.53^c^ ± 1.61		9.97^c^ ± 1.02	
PM baseline	7.84^a^ ± 1.76	0.015*	6.97^a^ ± 1.77	0.001*	7.06^a^ ± 1.15	0.001*
PM 1st month	8.22^ab^ ± 1.52		7.56^b^ ± 1.65		7.59^b^ ± 1.39	
PM 3rd month	8.50^b^ ± 1.48		8.16^c^ ± 1.55		8.06^c^ ± 1.36	

One-Way Repeated measures ANOVA and post-hoc Sidak tests; *p ≤ 0.05, **Means with different letters show significant difference. n: number of patients; SD: standard deviation; RLM: right lateral movement; LLM: left lateral movement; PM: protrusive movement.

**Fig. 5.** **Comparison of Lateral and Protrusive Movement values within and between groups.** (a) Comparison of Right Lateral Movement values within and between groups; (b) Comparison of Left Lateral Movement values within and between groups; (c) Comparison of Protrusive Movement values within and between groups. Group 1: Patients treated with OS, shown in blue. Group 2: Patients treated with OS and lidocaine injection to masseter muscle TPs, shown in red. Group 3: Patients treated with lidocaine injection to masseter muscle TPs, shown in green. Group 4: Healthy volunteers (control group), shown in orange. *p  ≤ 0.05. A statistically significant increase was observed in lateral and protrusive movements in treatment groups from baseline to 1st month and from 1st to 3rd month. In Group 3, right lateral movement increased from 1st to 3rd month but it was not statistically significant.

In Group 3, the RLM increased significantly in the 1st month (*p* = 0.001), but in the 3rd month it was not statistically more than the 1st month. In Group 1, the PM increased statistically from baseline to 3rd month (*p* = 0.015).

The RLM, LLM and PM in all 3 treatment groups were lower than the control group at baseline (*p* < 0.001, *p* < 0.001, *p* = 0.05, respectively). However, in 3rd month, all values increased and there was no statistically significant difference among the groups (*p *> 0.05).

There was no statistically significant difference in the amount of increase in RLM, LLM and PM between the groups from baseline to 1st month, from 1st month to 3rd month, and from baseline to 3rd month (*p *> 0.05).

### 3.4 Shear wave elastography

All stiffness values are shown in Table 6. There was no statistically significant difference between initial right and left masseter muscle stiffness in 3 treatment groups, and it was statistically higher than the control group. In control group, right and left masseter muscle stiffnesses were measured as 5.09 ± 0.93 kPa, 5.08 ± 0.83 kPa, respectively. Muscle stiffness decreased statistically significant from baseline to 1st month and from 1st to 3rd month in all treatment groups (*p* = 0.001) (Table 6). At the end of the treatment, there was no statistically significant difference between the right and left masseter muscle stiffness of the patients and the control group (*p* = 0.409 and *p* = 0.174, right and left respectively).

**Table 6.** Statistical comparison of the right and left masseter muscle stiffness within the groups.

Masseter muscle stiffness	Group 1 (n = 16)		Group 2 (n = 16)		Group 3 (n = 16)	
	Mean** ± SD (kPa)	*p*	Mean** ± SD (kPa)	*p*	Mean** ± SD (kPa)	*p*
R baseline	8.67^a^ ± 2.45	0.001*	8.42^a^ ± 2.61	0.001*	8.41^a^ ± 1.42	0.001*
R 1st month	6.53^b^ ± 1.67		6.24^b^ ± 1.91		6.36^b^ ± 1.15	
R 3rd month	5.47^c^ ± 1.48		4.81^c^ ± 1.00		5.29^c^ ± 1.06	
L baseline	8.93^a^ ± 2.44	0.001*	8.41^a^ ± 2.14	0.001*	8.36^a^ ± 1.35	0.001*
L 1st month	6.50^b^ ± 1.43		6.85^b^ ± 2.77		6.06^b^ ± 1.48	
L 3rd month	5.38^c^ ± 1.19		4.56^c^ ± 1.06		5.30^c^ ± 1.39	

One-Way Repeated measures ANOVA and post-hoc Sidak tests; *p ≤ 0.05, **Means with different letters show significant difference; n: number of patients; SD: standard deviation; R: right; L: left.

Right and left masseter muscle stiffness in all treatment groups decreased significantly at the end of the treatment. Periodic differences in right and left masseter muscle stiffness were not statistically significant among groups (Fig. 6a,b).

**Fig. 6.** **Comparison of masseter muscle stiffness values within and between groups.** (a) Comparison of right masseter muscle stiffness values within and between groups; (b) Comparison of left masseter muscle stiffness values within and between groups. Group 1: Patients treated with OS, shown in blue. Group 2: Patients treated with OS and lidocaine injection to masseter muscle TPs, shown in red. Group 3: Patients treated with lidocaine injection to masseter muscle TPs, shown in green. Group 4: Healthy volunteers (control group), shown in orange. *p  ≤ 0.05. Muscle stiffness decreased from baseline to 1st month and from 1st to 3rd month in all treatment groups.

Correlation between initial outcomes of masseter muscle shear wave elastography values and clinical findings revealed a significant positive correlation between right and left masseter muscle stiffness and VAS values (*R* = 0.558, *p* < 0.001, *R* = 0.597, *p* < 0.001), and a significant negative correlation between the amount of RLM and LLM (*R* = −0.341, *p* = 0.006, *R* = −0.340, *p* = 0.006). There was a significant positive correlation between left masseter muscle stiffness and pain free MO (*R* = 0.356, *p *= 0.45).

## 4. Discussion

Myofascial pain is a common condition that is gradually increasing in the population. There are some treatment modalities reported to be effective in the management of myofascial pain. In this study, pain-reducing effect of lidocaine injection to masseter muscle TPs was higher than the other treatments. The increase in MMO was higher in the OS and lidocaine injection combination. In all groups, masseter muscle stiffness decreased in correlation with clinical symptoms in patients with myofascial pain at the end of the study. The progress in clinical data was supported by the imaging findings, shear-wave sonoelastography.

The effect of OS therapy is controversial in the literature. In a six-week follow-up study, it is reported that in patients with myofascial pain, OSs reduced VAS scores and the number of sore muscles [16, 17]. In a study comparing behavioral therapy treatment and splint treatment in patients with myofascial pain, it was stated that both treatments were effective in reducing pain, but behavioral therapy treatment was more successful in the long term [18].

In one study, comparison of OS therapy alone and OS therapy combined with lidocaine injection into masseter, temporal and lateral pterygoid muscles for 3 months showed that pain decreased significantly in both groups, but the reduction was greater in the combination treatment group [14]. Contrary to that, in our study there was no difference regarding the effects of pain reduction between these two treatments. It may be due to the fact that only the TPs of the masseter muscle were injected in our study.

OS therapy has been reported to be successful in the treatment of TMD [14, 19, 20, 21, 22]. In a study comparing the OS treatment and the combined treatment of OS and TP injections, it has been reported that the use of OS, reduced pain and the number of TPs, but the combined treatment resulted in a greater reduction in VAS scores. It was also stated that TP lidocaine injection reduced pain more rapidly and shortened the duration of treatment [14].

The success of dry needling and lidocaine injection on pain relief in patients with myofascial pain was compared, and lidocaine injection was found to be more effective than dry needling [23]. There was no significant difference between success of procaine injection and dry needling, and post-injection sensitivity was reported to be higher in dry needling compared to local anesthesia injections [24, 25, 26, 27, 28].

Due to the risk of ischemic necrosis, local anesthetic is used without the addition of vasoconstrictor agent in TP treatment [1]. It is thought that repeated lidocaine injection applications will provide a more successful and long-lasting effect in reducing pain [29].

In a study comparing various combinations of arthrocentesis, OS and intramuscular injection treatments in patients with intra-articular irregularity and myofascial pain, it was stated that the effect of OS use was clearly observed in the 3rd month, while the effects of other treatments were seen in the 1st month [30]. Similar to the results of our study, it was concluded that when TP lidocaine injection and OS therapy are combined, it reduced pain in a shorter time compared to OS therapy alone.

In our study, injection of lidocaine into masseter muscle TPs treatment has shown a better pain reduction effect than OS and combination treatments. We think that the patient may perceive OS as a foreign body and this may cause grinding at night.

The pre-treatment painless unassisted MO of the OS and lidocaine injection treatment group was 35.81 ± 7.98 mm, and it was significantly lower than the control group. The amount of MMO after the treatment increased significantly from the baseline to the 1st month and from the 1st to 3rd month in all treatment groups. The increase of MMO at the end of the 3rd month was significantly higher in the combination of OS and masseter muscle TP lidocaine injection treatment than the treatments applied individually.

As a result of OS treatment and combination of OS and lidocaine injection treatment, unassisted MMO increased by an average of 7.87 mm and 10.07 mm, respectively. In two different studies which OS treatment was applied in patients with myofascial pain, an increase of 10.02 mm and 7.4 mm in MMO was observed, respectively [31, 32]. Similar to our results, it has been shown by various researchers that MMO increases with the use of splints [33, 34].

In a study where OS treatment with lidocaine injection was compared with OS treatment alone, it was reported that MMO increased significantly in both groups after 3 months. It was reported that there was a greater increase in MMO as a result of combination of splint with lidocaine injection treatment, but the difference between the two groups was not statistically significant [14].

In our study, patients who received combination treatment with lidocaine injection and OS achieved 40 mm MMO at the end of treatment, which is considered a normal range in adults [14]. In another study, combination of acupuncture, OS and TP injection was applied in patients with myofascial pain, and similar results were obtained. It was stated that a normal/ideal mouth opening range was achieved after treatment [35].

Decrease of RLL and LLM can be due to intra-articular irregularity, structural disorder or muscle pain. Less than 8 mm RLM and LLM is considered insufficient [36]. In our study, RLM and LLM were lower in all groups, and PM was lower in the group that was treated with combination of OS and lidocaine injection. At the end of the treatment, it was determined that the amount of eccentric movements in all 3 treatment groups increased and there was no statistically significant difference among the groups.

The limitation of eccentric movements in patients with myofascial pain may be due to pain-induced self-reluctance to move the mandible [30]. In our study, as a result of the treatments, pain decreased, parallel to increase in the mandibular movements.

The muscle stiffness of the patients with myofascial pain was higher than the healthy individuals before the treatment. It was determined that painful, symptomatic muscles had a higher stiffness due to its higher elastography score. The mean masseter muscle stiffness decreased significantly at 1st and 3rd months, respectively. There was no statistically significant difference between the groups in the amount of decrease. No superiority of any treatment over the other in reducing muscle stiffness was detected. As a result of the treatments, there was no significant difference between the masseter muscle stiffness of the treatment groups and the control group.

Ultrasonography can detect changes in the viscoelastic properties of a tissue, and the correlation between muscle structure and function can be better understood [9, 10].

A systemic review regarding the use of sonoelastography in the evaluation of the masseter muscle in healthy individuals and patients with masseter muscle disorders noted that sonoelastography is a promising tool for the assessment of masseter muscle stiffness, but more research is needed. Same as with our findings, it has been reported that muscle stiffness increases in patients with TMD and this stiffness is correlated with the severity of TMD symptoms. However, it has been suggested that elastography can be used to characterize masticatory muscle disorders and to conduct studies on larger groups [37].

In a study conducted to examine the reliability of stiffness measurement using shear-wave sonoelastography in healthy volunteers and to investigate normal, non-painful masseter muscle stiffness values, it was reported that the use of shear-wave sonoelastography was appropriate and showed a high level of reliability. The mean stiffness of the masseter muscles at rest in healthy volunteers was determined as 42.82 ± 5.56 kPa [38].

Thyroid gland, parotid gland, submandibular gland and masseter muscle stiffness were investigated in healthy adults and it was reported that masseter muscle stiffness was 10.4 ± 3.7 kPa. Further studies should be performed comparing the elasticity values of normal and pathological tissues in order to determine the diagnostic role of this technique [38]. In another study examining similar anatomical structures and the masseter muscle, the mean masseter muscle stiffness was determined as 10.0 ± 4.3 kPa [39].

In a study investigating the mean stiffness values of the temporomandibular joint disc and masseter muscle of 160 healthy adults, the mean stiffness values were determined as 15.17 ± 8.35 kPa for closed mouth and 15.87 ± 8.25 kPa for open mouth. There is no significant gender difference in the mean stiffness values of the masseter muscle. It has been reported that shear-wave sonoelastography is a useful imaging method that can be used together with routine ultrasonography in the evaluation of the temporomandibular joint disc and masticatory muscles [40]. It has been reported that shear-wave sonoelastography may be an indicator of muscle stiffness in patients with myofascial pain, and these patients may have a higher modulus of elasticity than healthy individuals [4, 5]. The presence of muscle tension or increased muscle stiffness in painful muscles is frequently reported in clinical practice [6, 7].

In patients with unilateral myofascial pain, muscle stiffness was compared with the contralateral side and healthy volunteers using strain sonoelastography. It has been reported that the symptomatic side has a higher masseter muscle sonoelastography value and this pain may be associated with increased muscle stiffness [38].

Right and left masseter muscle stiffnesses of healthy volunteers participating in our study were 5.09 ± 0.93 kPa and 5.08 ± 0.83 kPa, respectively. The highest mean value measured before the procedure in myofascial pain groups was 8.93 ± 2.44 kPa. These masseter muscle stiffness values are lower than the masseter muscle stiffness values in other studies. Since only patients with TMDs and myofascial pain were included in our study, muscle stiffness scores may be lower than the other studies.

It should also be noted that all of the values obtained from our study and reported in the literature are within the limits which can be considered as soft. However, there is a relativity for hardness values. In a study evaluating liver US elastography, it was reported that when the body mass index is below 25 kg/m^2^ and the hardness value is below 7.1 kPa in the first measurement, a single measurement may be sufficient and an approach in which 3 measurements are made and the average is taken is not superior to such an approach [41]. Masseter is a superficially localized muscle, so it can be predicted that body mass index may be less effective in masseter measurements compared to liver. We can claim that the elastography scores we obtained for masseter muscle, which are lower than the averages reported in the literature, can be reliable. In addition, technically, sufficient window filling was obtained during the measurements and a homogeneous color map was obtained. During the examination, situations such as muscle tightening or pressure with a probe, which may cause false high values, were prevented. When the subcutaneous adipose tissue is too thin and there is a concern that a reliable measurement cannot be made, a thick gel pad was formed between the probe and the skin. Therefore, it can be understood that the lower values obtained from our study compared to the literature are reliable and result from the accuracy in the measurement technique.

In our study, a significant positive correlation was found between masseter muscle stiffness and VAS value. Also a significant negative correlation was found between masseter muscle stiffness and the amount of lateral movement. This is in line with studies stating that when the severity of TMD symptoms of patients increases, muscle stiffness also increases [37]. When patients’ pain increases, it is expected that there will be restriction in mandibular lateral movements. One limitation of this study is that we did not evaluate patient self-assessment of the treatment outcome.

Shear wave sonoelastography has been recently used in the assessment of TMD and myofascial pain treatment outcomes. There are few studies regarding evaluation of the masseter stiffness with shear wave elastograph following conservative therapy, but randomized controlled studies were lacking. We aimed to fill this gap in the literature with a randomized and controlled study of the effects and comparisons of the most commonly used methods in patients with myofascial pain.

The temporal muscle, which is one of the masticatory system muscles, is a very superficial muscle and has a very thin and scalped skin. Investigation of this muscle was not planned at the beginning of the study because it was thought that sonoelastographic measurement would be difficult and safe results would not be obtained. The patients in Groups 2 and 3 were given two additional appointments for masseter muscle injections on the 7th and 14th days, different from the appointments of the patients in Group 1. We did not even out the recall frequency among the groups. The impact of equal recalls for all the treatment groups is obscure. Behavioral suggestions were given to each of the groups and training was given in this direction. Therefore, a separate behavioral treatment group has not been established. A behavioral therapy group may be included in similar studies in the future. In this study, 3-month results were examined. Studies with longer follow-up periods can be planned with larger patient groups.

## 5. Conclusions

In our study, OS, lidocaine injection of masseter muscle TPs and combination treatments were applied to patients with muscle-induced TMD and myofascial pain. It was determined that all patients showed clinical progress. Pain decreased, MMO values increased and masseter muscle stiffness decreased in all patients. It was observed that all treatment protocols have successful results, but the advantages of the treatments on different clinical parameters are different.

## 6. Key findings

• The analgesic effect of masseter muscle TP lidocaine injection was higher than OS and combination treatment.

• It was determined that the combination treatment of OS and lidocaine injection showed superior results in increasing the MMO.

• At the end of all the treatments, the symptoms of the patients improved, including decrease in VAS scores, increase in the MMO and increase in the amount of eccentric motion.

• Masseter muscle stiffness decreased following all treatments. The correlation between muscle stiffness and clinical symptoms was also shown and supported by shear-wave sonoelastography, ultrasonography.
